# Septic Pulmonary Embolism Secondary to* Klebsiella pneumoniae* Epididymitis: Case Report and Literature Review

**DOI:** 10.1155/2019/5395090

**Published:** 2019-03-19

**Authors:** Juan Sebastián Alonso Ojeda Gómez, Jorge Alberto Carrillo Bayona, Laura Cristina Morales Cifuentes

**Affiliations:** Hospital Universitario Mayor Méderi, Universidad del Rosario, Bogotá, Colombia

## Abstract

**Background:**

Septic pulmonary embolism (SPE) is defined as the occurrence of septic thrombi in the pulmonary circulation. We report a case of SPE secondary to* K. pneumoniae* epididymitis.

**Case Presentation:**

A 74-year-old male with a history of diabetes mellitus experienced SPE secondary to epididymitis, with isolation of* K. pneumoniae* in blood and presence of lung nodules, with a chest computed tomography showing the halo and reversed halo signs.

**Discussion:**

SPE is characterized by the presence of septic thrombi in the pulmonary circulation coming from an extrapulmonary infective focus. SPE secondary to K. pneumoniae epididymitis is an uncommon condition that is characterized by the presence of multiple bilateral nodules of peripheral distribution.

**Conclusion:**

SPE is an unusual complication of acute epididymitis. Suspicion of SPE should be considered in patients with a diagnosis of epididymitis, respiratory symptoms, and multiple nodules in chest imaging assessments.

## 1. Background

Septic pulmonary embolism (SPE) is defined as the occurrence of septic thrombi in the pulmonary circulation coming from a primary extrapulmonary infective focus [1]. Epididymitis is an inflammation of the epididymis that is mostly caused by an infection derived from the bacterial infection of the urinary tract. Epididymitis is most commonly unilateral and, according to the age group, relates to uropathogens or sexually transmitted microorganisms. Clinically, the patients present epididymal pain or palpation and scrotal edema [2, 3]. We report a case of SPE secondary to* Klebsiella pneumoniae *epididymitis.

## 2. Case Presentation

**Figure 1:**
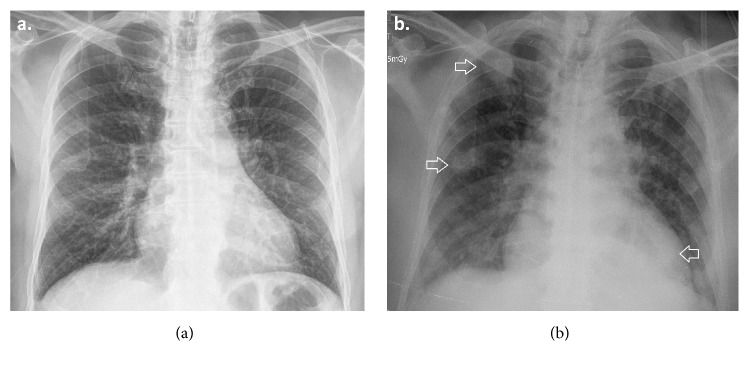
Chest X-ray. (a) Thickening of bronchial walls with no concomitant parenchymal abnormalities. (b) Multiple ill-defined lung nodules (arrows). Enlargement of the cardiac silhouette and left pleural fluid.

**Figure 2:**
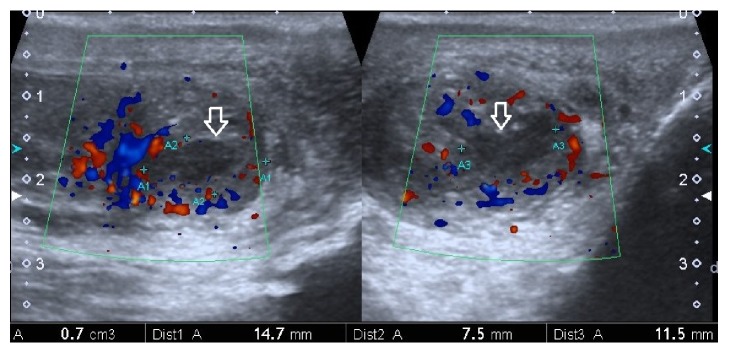
US Testicular Doppler. Hypoechoic collection of 0.7 cm^3^ with debris in its interior (arrow) located in the tail of the left epididymis associated with an increase in volume and vascularization of the epididymis (blue and red signal).

A 74-year-old male patient was admitted to our institution with a one-month left-sided testicular pain and occasional dry cough, unquantified fever, asthenia, adynamia, and decreased appetite in the last four days. His history included insulin-requiring type 2 diabetes mellitus, arterial hypertension, stage 3 chronic renal disease, and chronic obstructive pulmonary disease. On physical examination, the patient was febrile and tachycardic and had left testicular edema with pain on epididymal palpation. An initial evaluation consisted of laboratory tests that showed neutrophilic leukocytosis (WBC 24.2 x10^3^/uL, neutrophils 87%). Chest X-ray evidenced thickening of the bronchial walls with no concomitant parenchymal abnormalities (Figure 1(a)) and urinalysis showed an increase in white blood cells and few Gram-negative bacteria. The testicular ultrasound showed an abscess in the tail of the left epididymis (Figure 2); oral doxycycline therapy was administrated.

**Figure 3:**
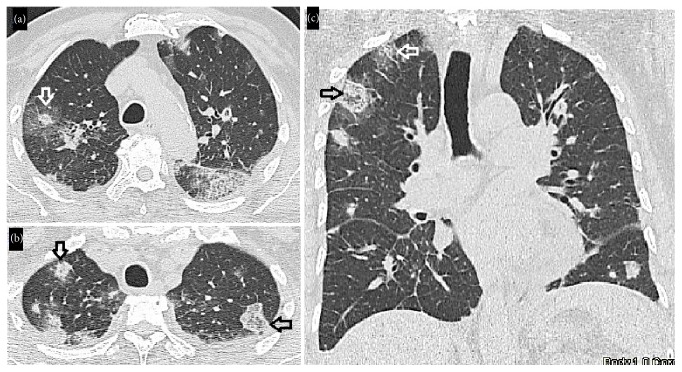
Chest CT scan. (a) Consolidations with peripheral ground-glass opacities compatible with halo sign in the apical segments of the upper lobes (white arrow). (b) Nodule with central ground-glass density and consolidation ring (reversed halo sign) in the apical segment of the upper lobes (black arrow). (c) Coronal reconstruction. Multiple peripheral nodules, some of them with reversed halo sign (black arrow) and halo sign (white arrow).

**Figure 4:**
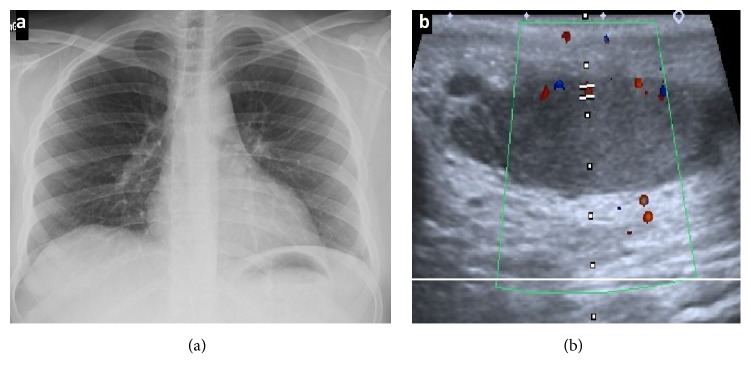
Chest X-ray. (a) There are no consolidations in the lung parenchyma or pleural effusion. US Testicular Doppler. (b) Testicle and left epididymis of normal size and vascularization.

On the fourth day, the patient developed clinical deterioration with worsening of respiratory symptoms and inadequate metabolic control (HbA1c 8.7% and central glycemia 510 mg/dL) requiring transfer to the intermediate-care unit for management of hyperosmolar hyperglycemic state. The computerized tomography (CT) of the abdomen and pelvis was normal but a follow-up chest X-ray evidenced multiple bilateral nodular lesions and left pleural effusion (Figure 1(b)) and positive blood and urine cultures yielding* K. pneumoniae* sensitive to penicillin, quinolone, and carbapenem. The CT of the chest confirmed the presence of multiple bilateral nodules with halo sign and reversed halo sign (Figure 3). Presence of an extrapulmonary infective focus along with respiratory symptoms, isolation of bacteria in blood, and multiple bilateral nodules in the chest CT scan led to considering the diagnosis of SPE secondary to epididymitis. Therefore, it was decided to suspend the doxycycline and start intravenous ciprofloxacin. Clinical evolution after a 14-day antibiotic course was satisfactory with an improvement of the respiratory symptoms and marked decrease in pain and testicular edema, the chest X-ray at day 14th showed a disappearance of the consolidations, and the testicular ultrasound of follow-up at day 16th showed a decrease in local inflammatory signs and resolution of the abscess in the tail of the epididymis (Figure 4).

## 3. Discussion

SPE is defined as a nonthrombotic pulmonary embolism secondary to the presence of septic aggregates in the pulmonary circulation from an extrapulmonary infective focus. The obstruction of blood flow within small pulmonary artery vessels can result in lung infarction. On the other hand, the presence of microorganisms within the thrombus induces an inflammatory reaction which may trigger focal abscess formation [4, 5].

There are several conditions associated with SPE, including intravascular device-related infections, infective endocarditis, liver or kidney abscess, infections of the skin and soft tissues, septic thrombophlebitis, Lemierre's syndrome, use of intravenous drugs, and periodontal infection [5–10].

Diagnosis of SPE is based on the combination of clinical and imaging findings. Clinical manifestations of SPE are nonspecific and depend on the primary infective focus. The most common symptoms include fever, dyspnea, cough, pleuritic chest pain, and hemoptysis [1, 4]. Microorganisms found in association with SPE include* Staphylococcus aureus, *coagulase-negative* Staphylococcus, Streptococcus spp., Fusobacterium, *and* K. pneumoniae* [1, 4, 5].

Chest X-ray is the first line of diagnostic imaging for the assessment of patients suspected of SPE. The most relevant radiographic abnormality in SPE is the presence of nodules. In most cases, nodules are multiple with a peripheral distribution and variable size and, in some cases, show a radiolucent center suggesting cavitation. Other findings described include consolidation, lymph node enlargement, and pleural effusion [1]. CT scan is currently considered the method of choice for evaluating patients suspected of SPE [5].

Findings on CT scans include bilateral nodules (82%), cavitation (55%), pleural effusion (29%), afferent vessel sign (27%), and parenchymal cuneiform opacities (17%). In patients with SPE, the halo sign has also been described [4].

Epididymitis is the inflammation of the epididymis and can be associated with both infectious and noninfectious conditions. Epididymitis can be classified as acute or chronic according to the duration of symptoms (less than 6 weeks in acute forms). Bacterial infections constitute the most common cause of acute epididymitis [2].

Overall, epididymitis in sexually active men under 35 years is unilateral and related to sexually transmitted microorganisms and, in older men, it is secondary to uropathogens [3]. Clinical manifestations include gradual onset of testicular pain with unilateral radiation to the hypogastrium and scrotal edema. Other symptoms include urethral secretion, dysuria, urgency, scrotal erythema, and fever [2].

Imaging studies in our patient showed nodules with the reversed halo sign. The reversed halo sign is defined as a ground-glass area surrounded by a consolidation ring [11]. This has been initially identified in cryptogenic organizing pneumonia. However, in granulomatous diseases such as granulomatosis with polyangiitis (previously known as Wegener's granulomatosis), pulmonary tuberculosis, sarcoidosis, and nongranulomatous invasive fungal infections such as bronchogenic cancer and pulmonary infarction the reversed halo sign has also been described [11–13].

A systematic literature search in the following databases: Pubmed, Embase, ScienceDirect, Lilacs, Proquest, Google Scholar, and gray literature (OPAC, Opensigle, Teseo, TripDataBase) using the MESH terms: “septic pulmonary embolism AND halo sign”, “septic pulmonary emboli AND halo sign”, “septic pulmonary emboli AND reverse halo sign”, “septic pulmonary emboli AND epididymitis”, “pulmonary emboli AND epididymitis” with no limits on publication year or language was conducted. Case series reporting an occurrence of halo sign in patients with SPE were found [9, 14]. In the study by Kwon et al. [14], the halo sign and the afferent vessel sign occurred more commonly in cases of SPE caused by Gram-negative bacteria, whereas the presence of cavitation and air bronchogram was more common in SPE caused by Gram-positive bacteria. In addition, Cheng et al. [15] reported a single case of SPE secondary to* K. pneumoniae* epididymitis.

The similar features shared by our patient and the case reported by Cheng et al. [15] include epididymitis with the isolation of* K. pneumoniae*, multiple bilateral nodular lesions with pleural effusion, and history of diabetes mellitus. The study conducted by Lee et al. [16] evaluated the impact of an environment with high glucose concentrations on* K. pneumoniae *infections. The authors concluded that high glucose concentrations surrounding the bacteria decrease phagocytosis and bactericidal activity of neutrophils, conditioning an increased synthesis of bacterial polysaccharide capsule. Extrapolating the results in patients with uncontrolled diabetes mellitus (HbA1c > 7% and glycemia >200), this represents an independent risk factor for the occurrence of invasive infections with* K. pneumoniae*.

## 4. Conclusion

SPE is an uncommon complication of acute epididymitis. The case described is the second case reported in the literature. SPE should be considered in the differential diagnosis of patients presenting extrapulmonary infective focus, respiratory symptoms, and parenchymal pulmonary nodules on imaging studies of the chest.

## Abbreviations

SPE: Septic pulmonary embolism
CT: Computerized tomography.
